# Isolated Self-Limited Right Oculomotor Nerve Palsy With Positive Asialo-GM1 Antibody After SARS-CoV-2 mRNA Vaccination

**DOI:** 10.7759/cureus.65045

**Published:** 2024-07-21

**Authors:** Ifeanyichukwu Ozobu, Ethan Salter, Sophia Salter, Davin Peng, Arash Sherbaf, Arvind Ravinutala, Antonio K Liu

**Affiliations:** 1 Neurology, Adventist Health White Memorial, Los Angeles, USA; 2 Internal Medicine, Adventist Health White Memorial, Los Angeles, USA; 3 Neurology, Loma Linda University School of Medicine, Loma Linda, USA

**Keywords:** oculomotor nerve (cn iii) palsy, moderna, pfizer, covid-19, asialo-gm1 antibody

## Abstract

mRNA vaccines have been a critical tool in combating the current coronavirus disease 2019 (COVID-19) pandemic and demonstrated a high safety profile. However, rare cases of isolated oculomotor nerve palsy following vaccination have been reported. These few reported cases can be divided into two groups based on symptom onset: immediate and delayed. While most reported cases involving Pfizer and Moderna vaccines occurred within the first few days of vaccination, a few cases with delayed onset have also been described. We present a unique case of a patient experiencing isolated, unilateral oculomotor nerve palsy 14 days after receiving a Moderna booster shot. Notably, our case and a previously reported case of 17-day onset case share the interesting finding of positive ganglioside antibodies. This not only highlights the potential for unusual occurrences following COVID-19 vaccination but also opens up avenues for exploring the underlying mechanisms behind these events.

## Introduction

Oculomotor nerve palsy, also known as third nerve palsy, results from damage to the third cranial nerve. This damage causes characteristic symptoms, such as a drooping eyelid, double vision, pupil dilation, and deficits in adduction and vertical gaze. Pupil involvement in oculomotor palsy can differ depending on the underlying condition, resulting in pupil-sparing or non-pupil-sparing outcomes. Some conditions, like diabetes mellitus and hypertension, often lead to pupil-sparing palsy [1,2]. Conversely, compressive lesions or damage in the midbrain can cause non-pupil-sparing palsy, but these are less common. This report will focus on pupil-sparing cases.

The risk of oculomotor palsy increases with age, occurring most commonly in patients between 70 and 90 years of age, with an annual incidence of 3.71-4.0 per 100,000 [3]. There are several potential causes for oculomotor palsy: microvascular, vascular anomalies; vascular, inflammatory, infectious, traumatic, neoplastic, and congenital; recurrent painful ophthalmoplegic neuropathy; central demyelination; iatrogenic and idiopathic; Guillain Barre syndrome (GBS); and Miller Fisher syndrome (MFS) [2]. Of these, microvascular causes are the most frequent (26.5% of cases), followed by vascular anomalies (17.4%), neoplastic (13.6%), inflammatory (12.5%), idiopathic (9.5%), and traumatic (8.4%). Interestingly, these etiologies are linked to certain demographic differences, with microvascular being the most common cause of oculomotor palsy in men and vascular anomalies being the most common in women [2].

Several studies have reported cases of multiple cranial nerve palsies following coronavirus disease 2019 (COVID-19) vaccination [4]. These studies have investigated the characteristics of these cranial nerve palsies with a focus on the most commonly affected nerves. The optic (CN 2) and facial (CN 7) nerves appear to be the most susceptible, while the involvement of the oculomotor (CN 3), trochlear (CN 4), and abducens (CN 6) nerves have also been documented, both in isolation and in combination. Interestingly, isolated oculomotor nerve palsy following the SARS-CoV-2 mRNA vaccination seems to be a rare occurrence. We discuss an uncommon presentation of an isolated oculomotor nerve palsy following the SARS-CoV-2 mRNA vaccination.

Asialo-GM1 antibody, a type of ganglioside antibody, has garnered significant attention in the field of autoimmune diseases. Gangliosides are glycosphingolipids present in the outer layer of cells throughout the body with a particular abundance in nerve tissues, where they compose 6% of all lipids. While the immune system typically functions to defend against foreign invaders, the presence of Asialo-GM1 antibodies indicates a potential malfunction. These antibodies can mistakenly target the body's own gangliosides, potentially triggering a destructive immune response that underlies various neurological autoimmune pathologies. Hence, Asialo-GM1 antibodies function as an indicator of autoimmunity. Notably, Asialo-GM1 antibodies have been found in a variety of neurological conditions, such as Parkinson’s disease, Alzheimer’s disease, multiple sclerosis, MFS, and GBS [5]. This case report explores the potential association between Asialo-GM1 antibodies and oculomotor nerve palsy following the SARS-CoV-2 mRNA vaccination.

## Case presentation

An 81-year-old male presented to the emergency department with two days of diplopia. While he had no prior confirmed COVID-19 infection, he had received a Moderna SARS-CoV-2 mRNA booster vaccination 16 days before the onset of diplopia (onset of symptoms occurred 14 days after the vaccination). He only had mild malaise the day after the shot. His past medical history, family history, and social history were all non-contributory.

**Figure 1:**
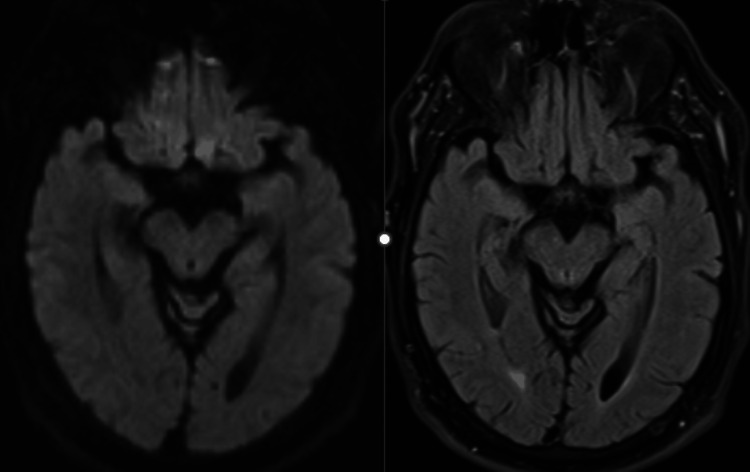
MRI DWI (right) and FLAIR (left) sequences of the midbrain showed no abnormality DWI: diffusion-weighted imaging; FLAIR: fluid-attenuated inversion recovery; MRI: magnetic resonance imaging

Upon examination, the patient was pleasant, coherent, cooperative, and oriented. On cranial nerve examination, he had a right ptosis and deficiencies in right eye adduction and vertical gaze. His pupils were both reactive to light. On digital pupillometry, his pupils showed slightly different sizes (right: 3.22 mm, left: 3.45 mm) and demonstrated decreased reactivity in the right eye (Neurological Pupillary Index: right 2.2, left 2.7). These findings were consistent with an isolated right oculomotor nerve palsy that spared the pupil. The rest of his neurological exam was normal, including his motor function, sensation, balance, and gait examination. CT and MRI of the head were negative (Figure 1). Although the patient refused a lumbar puncture, various blood tests were performed to check for myasthenia gravis, autoimmune neurological diseases, and ganglioside antibodies.

Fortunately, the patient's condition started to improve on the third day in the hospital without needing any immunosuppressive medications. He was discharged home shortly after. Subsequently, the only antibody that came back positive was serum Asialo-GM1 antibodies, IgG/IgM 70 IV (0-50). A follow-up appointment 14 days after discharge confirmed the complete resolution of all his symptoms.

## Discussion

Our patient presented with isolated pupil-sparing oculomotor nerve palsy, suggesting that a general neuromuscular junction problem or descending demyelination etiology was unlikely. Carefully obtained history revealed no other preceding event except for his receiving the Moderna booster vaccination 14 days prior. Together with a positive Asialo-GM1 antibody, this suggested a possible post-vaccination autoimmune reaction. However, the lack of CSF limited our ability to confirm this.

Our literature search yielded seven case reports describing confirmed isolated oculomotor nerve palsy [6-12]. We also identified a few case series. However, after excluding duplicate reports, only two case series were pertinent [13,14]. One series reported a single additional case of oculomotor nerve palsy [13]. The other series included nine additional cases [14], but lacked specific details on their clinical features, making it impossible to confirm if these truly represented isolated oculomotor palsy. Including our patient, we examined nine total cases of confirmed isolated oculomotor nerve palsy. Three of the cases had received the Moderna vaccine while six had received the Pfizer vaccination. The onset of symptoms varied: six patients experienced them within one to six days post-vaccination (three on day one, two on day three, and one on day six). In addition, three patients developed symptoms later, at days 12, 14 (our case), and 17 (two with Moderna and one with Pfizer).

Two main mechanisms have been proposed to explain the onset of oculomotor nerve palsy following COVID-19 vaccination. The first suggests direct damage to the nerve due to an immune response triggered by the vaccine itself [9,15]. This mechanism might involve interferons, similar to what has been observed in interferon-mediated Bell's palsy after COVID-19 vaccination [16]. The second theory proposes an indirect immune-mediated injury to the nerve. In this scenario, the body's inflammatory response to the vaccine triggers an attack on the nerve, potentially leading to demyelination or reduced blood flow and ultimately oculomotor nerve palsy. Other established causes of oculomotor nerve palsy exist, such as hyperviscosity leading to nerve ischemia [17]. These typically present more immediately and might explain the cases with rapid onset. However, for delayed-onset cases like our patient, molecular mimicry might be the underlying mechanism. This autoimmune mechanism involves the immune system mistaking the body's tissues for a foreign invader. Autoimmune neurological conditions like GBS and MFS share this delayed presentation and underlying mechanism [18,19].

Interestingly, of the nine cases analyzed, the two patients with delayed presentations (after 12 and 17 days) who received plasma exchange therapy (a type of immunosuppressive therapy used to rapidly remove autoantibodies) recovered successfully. Similar to our patient, the patient whose symptoms presented after 17 days had positive anti-GQ1B antibodies, a marker of autoimmunity. Our patient's positive Asialo-GM1 antibodies, another marker of autoimmunity, along with the delayed presentation, suggest an autoimmune etiology. However, the rapid improvement our patient experienced indicated that immunosuppressive therapy was not necessary. Overall, these findings suggest that the cause of the oculomotor nerve palsy presentation after COVID-19 vaccination, especially in the delayed-onset group, may have an autoimmune origin.

## Conclusions

This case report presented an 81-year-old male who developed isolated, pupil-sparing oculomotor nerve palsy 14 days after receiving a Moderna COVID-19 booster vaccination. The positive Asialo-GM1 antibody test suggests a possible post-vaccination autoimmune reaction, but the limitations intrinsic to a single case report mean we are unable to draw definitive conclusions about the causality. Further research is needed to investigate the potential link between COVID-19 vaccination and isolated oculomotor nerve palsy.

Four years into the COVID-19 pandemic, our understanding of the virus and the effects of vaccination has significantly improved. It is important to emphasize that vaccination programs have demonstrably saved countless lives and vaccines remain overwhelmingly safe. However, rare complications, such as isolated oculomotor deficits, may still occur. These cases highlight the possibility of an autoimmune response as a contributing factor. Therefore, practitioners should consider immunosuppressive therapy as a treatment option for patients whose condition does not improve.
